# Role of Damage Control Surgery in the Treatment of Hinchey III and IV Sigmoid Diverticulitis: A Tailored Strategy

**DOI:** 10.1097/MD.0000000000000184

**Published:** 2014-11-28

**Authors:** Roberto Cirocchi, Alberto Arezzo, Nereo Vettoretto, Davide Cavaliere, Eriberto Farinella, Claudio Renzi, Gaspare Cannata, Jacopo Desiderio, Federico Farinacci, Francesco Barberini, Stefano Trastulli, Amilcare Parisi, Abe Fingerhut

**Affiliations:** From the Department of Digestive Surgery (RC, JD, ST, AP), St. Maria Hospital, University of Perugia, Terni; Department of Surgical Sciences (AA), University of Turin, Turin; Laparoscopic Surgical Unit (NV), M. Mellini Hospital, Chiari; Department of Surgical Oncology (DC), Forlì; Department of HPB and Digestive Surgery (EF), Ospedale Mauriziano Umberto I, Turin; Department of General and Oncologic Surgery (CR, GC, FB), University of Perugia, Perugia; Department of Mininvasive and Robotic Surgery (FF), St. Maria Hospital, University of Perugia, Terni, Italy; Athens First Department of Surgery (Prof Leandros) (AF), Hippokration University Hospital, University of Athens, Athens, Greece; and Section for Surgical Research (Prof Uranues) (AF), Department of Surgery, Medical University of Graz, Graz, Austria

## Abstract

Many of the treatment strategies for sigmoid diverticulitis are actually focusing on nonoperative and minimally invasive approaches. The aim of this systematic review was to evaluate the actual role of damage control surgery (DCS) in the treatment of generalized peritonitis caused by perforated sigmoid diverticulitis.

A literature search was performed in PubMed and Google Scholar for articles published from 1960 to July 2013. Comparative and noncomparative studies that included patients who underwent DCS for complicated diverticulitis were considered.

Acute Physiology and Chronic Health Evaluation score, duration of open abdomen, intensive care unit length of stay, reoperation, bowel resection performed at first operation, fecal diversion, method, and timing of closure of abdominal wall were the main outcomes of interest.

According to the Preferred Reporting Items for Systematic Reviews and Meta-Analyses algorithm for the literature search and review, 10 studies were included in this systematic review. DCS was exclusively performed in diverticulitis patients with septic shock or requiring vasopressors intraoperatively. Two surgical different approaches were highlighted: limited resection of the diseased colonic segment with or without stoma or reconstruction in situ, and laparoscopic washing and drainage without colonic resection.

Despite the heterogeneity of patient groups, clinical settings, and interventions included in this review, DCS appears to be a promising strategy for the treatment of Hinchey III and IV diverticulitis, complicated by septic shock. A tailored approach to each patient seems to be appropriate.

## INTRODUCTION

Generalized peritonitis as a consequence of complicated acute diverticulitis (AD) is rare and corresponds to stages III and IV of the Hinchey classification.^1^ Mortality is still high, up to 40% of cases, despite the progress in antibiotics regimens and fluid administration.^2^ Hartmann procedure (HP) has long been considered to be a safe treatment for this severe clinical condition, accounting for about a half of all patients undergoing surgery for complicated AD in Europe, mostly as an open procedure;^3^ however, more than 1/3 of the patients who undergo a HP do not have their stoma reversed within 1 year,^4^ if ever^5^ and when they have, they are exposed to the risk of a difficult surgical procedure due to the unrare sequela of the generalized peritonitis. Combined with improvements in medical support, this has led surgeons to consider whether resection with primary anastomosis (PA) or any other less-invasive surgical procedure could provide equivalent safety. Two multicenter trials,^6,7^ the latter aborted because of difficult accrual,^8^ found substantial equivalence between HP and PA groups in terms of morbidity, although patients with both purulent and fecal peritonitis were included. A recent systematic review and meta-analysis including 1041 patients, despite a vast heterogeneity of patients’ characteristics, suggested an advantage in terms of mortality and hospital stay in favor of PA.^9^

Patients with Hinchey III and IV with severe sepsis might benefit from damage control surgery (DCS).^10^ Initially described for the treatment of major abdominal injuries,^11,12^ indications for DCS have been extended to patients with necrotizing pancreatitis, severe peritonitis, or intraperitoneal hemorrhage.^13^

The aim of this systematic review was to evaluate the actual role of DCS in the treatment of generalized peritonitis caused by perforated sigmoid diverticulitis and determine whether there were any specific indications for one approach or another

## METHODS

A systematic literature search was performed on PubMed and Google Scholar from January 1960 to July 2014. The Preferred Reporting Items for Systematic Reviews and Meta-Analyses was followed. The following search strategies were used in PubMed:

1. damage[All Fields] AND (“prevention and control”[Subheading] OR (“prevention”[All Fields] AND “control”[All Fields]) OR “prevention and control”[All Fields] OR “control”[All Fields] OR “control groups”[MeSH Terms] OR (“control”[All Fields] AND “groups”[All Fields]) OR “control groups”[All Fields]) AND (“surgery”[Subheading] OR “surgery”[All Fields] OR “surgical procedures, operative”[MeSH Terms] OR (“surgical”[All Fields] AND “procedures”[All Fields] AND “operative”[All Fields]) OR “operative surgical procedures”[All Fields] OR “surgery”[All Fields] OR “general surgery”[MeSH Terms] OR (“general”[All Fields] AND “surgery”[All Fields]) OR “general surgery”[All Fields]) AND (“diverticulitis”[MeSH Terms] OR “diverticulitis”[All Fields])
2. damage[All Fields] AND (“prevention and control”[Subheading] OR (“prevention”[All Fields] AND “control”[All Fields]) OR “prevention and control”[All Fields] OR “control”[All Fields] OR “control groups”[MeSH Terms] OR (“control”[All Fields] AND “groups”[All Fields]) OR “control groups”[All Fields]) AND (“laparotomy”[MeSH Terms] OR “laparotomy”[All Fields]) AND (“diverticulitis”[MeSH Terms] OR “diverticulitis”[All Fields])
3. (“colon, sigmoid”[MeSH Terms] OR (“colon”[All Fields] AND “sigmoid”[All Fields]) OR “sigmoid colon”[All Fields] OR “sigmoid”[All Fields]) AND (“diverticulitis”[MeSH Terms] OR “diverticulitis”[All Fields]) AND damage[All Fields] AND (“prevention and control”[Subheading] OR (“prevention”[All Fields] AND “control”[All Fields]) OR “prevention and control”[All Fields] OR “control”[All Fields] OR “control groups”[MeSH Terms] OR (“control”[All Fields] AND “groups”[All Fields]) OR “control groups”[All Fields]) AND (“surgery”[Subheading] OR “surgery”[All Fields] OR “surgical procedures, operative”[MeSH Terms] OR (“surgical”[All Fields] AND “procedures”[All Fields] AND “operative”[All Fields]) OR “operative surgical procedures”[All Fields] OR “surgery”[All Fields] OR “general surgery”[MeSH Terms] OR (“general”[All Fields] AND “surgery”[All Fields]) OR “general surgery”[All Fields])
4. feculent [All Fields] AND (“peritonitis”[MeSH Terms] OR “peritonitis”[All Fields])

All titles and abstracts were assessed to select those focusing on DCS. Subsequently, the full text of the selected trials was independently screened by 2 authors (RC and GC) for eligibility.

When there was overlapping between multiple articles published by the same authors and no difference in the examined time, only the most recent trial was enclosed to avoid redundant counting. The PubMed function “related articles” were used to search further articles. The references of the included studies were evaluated for other potential trials missed by the screening process. Ethical approval for this study was not necessary because of its design consisting a systematic review of the literature.

### Inclusion Criteria

We considered both comparative and noncomparative studies, which included patients who underwent DCS for complicated diverticulitis irrespectively of their size, publication status, or language. Comparative studies were included if they focused on selected outcomes of interest (Acute Physiology and Chronic Health Evaluation [APACHE] II score, duration of open abdomen, intensive care unit [ICU] day, reoperation, bowel resection performed at first operation, fecal diversion, method, and timing of closure of abdominal wall), irrespective of the type of surgical approach used for comparative group (laparoscopic or open).

### Data Extraction

Included studies were reviewed and data were extracted by 2 blinded reviewers (RC and GC) using a standardized data extraction form.

### Assessment of Risk of Bias in Included Studies

We assessed the methodological quality of the trials independently, without masking the trial names. The review authors performed the risk of bias assessment according to “The Cochrane Collaboration tool for assessing risk of bias.”^14^

## RESULTS

**FIGURE 1:**
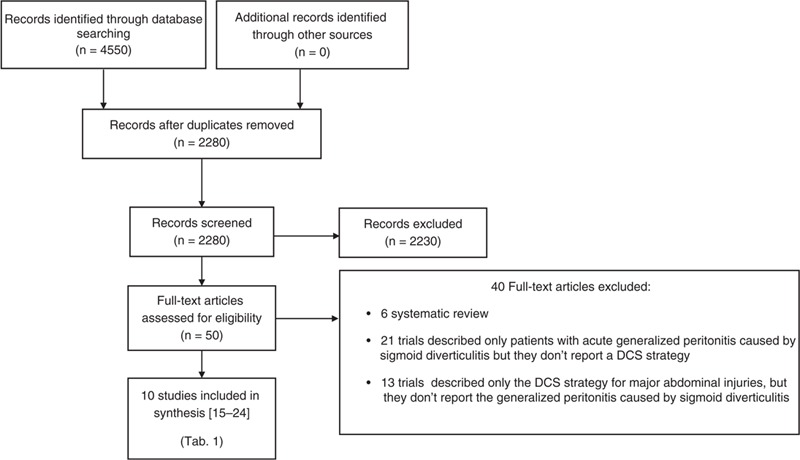
PRISMA flowchart of literature search. PRISMA = Preferred Reporting Items for Systematic Reviews and Meta-Analyses.

Our comprehensive literature search identified 4550 records, of which 50 full texts were identified for further examination, while the others were excluded based on the titles and/or abstract. According to the inclusion criteria, 10 studies were selected for the current review and analysis. All studies were published in English (Figure 1).

### Description of Studies

A detailed description of the characteristics of patients included in the 10 selected studies is presented in Table 1 .^15–24^

**TABLE 1:**
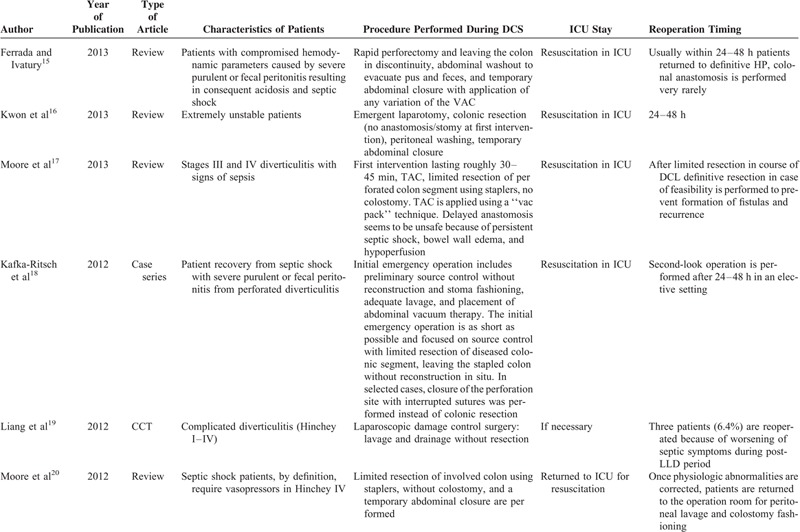
Characteristics of the Included Studies

**TABLE 1 (Continued):**
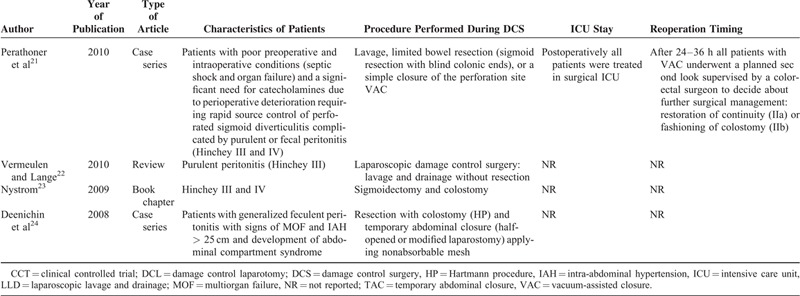
Characteristics of the Included Studies

Only patients with septic shock caused by severe purulent (Hinchey III) or fecal (Hinchey IV) peritonitis^15^ and who required catecholamine administration because of perioperative general conditions^15,16,20,21^ underwent conventional DCS.

### Definition of DCS

Two different DCS approaches have been described in the literature to treat acute peritonitis caused by diverticulitis.

The first, that is, conventional,^15,16,18,20,21,24^ is characterized by the shortest possible initial emergency operation focused on source control (limited resection of the diseased colonic segment) leaving the stapled remaining colon with or without a stoma or reconstruction in situ. In selected cases, instead of colonic resection, closure of the perforation site with interrupted sutures, adequate lavage, and temporary closure was performed. In all cases after 24–48 hours of reequilibration in the ICU, definitive surgical treatment was applied. The authors who followed this approach proposed different abdominal closure techniques: most recommended temporary abdominal closure while a few preferred vacuum-assisted closure (VAC).^15,21^

The second consists laparoscopic abdominal washing and drainage only, without resection.^19,24^ The proponents of washing and drainage^19,24^ considered these procedures as definitive surgery and reintervention was performed only in case of postoperative complications.^19^ Moore et al^20^ did not consider this technique as DCS and reserved it only for patients with Hinchey III without septic shock.

### Patients Who Underwent DCS

Conventional DCS^15,16,18,20,21,24^ was performed in patients with septic shock defined as hemodynamically unstable patients who required immediate inotropic or vasopressor support^15,16,20,21^ and generalized purulent or fecal peritonitis; on the contrary, patients who underwent laparoscopic DCS had only generalized purulent or fecal peritonitis and were not hemodynamically unstable.^19,22^ Kown et al^16^ recommended the HP in unstable but controllable patients, while DCS should be limited to extremely unstable patients.

DCS involved a 3-staged approach—stage I: an abbreviated initial operative procedure with temporary abdominal closure; stage II: continued resuscitation and management of physiologic and acid–base derangements; and stage III: definitive treatment and closure.^25^

Subgroup analysis for type of abbreviated initial operative procedure with temporary abdominal closure (stage I of DCS) revealed that the majority of surgeons insisted on limited resection of diseased colon. Both Kafka-Ritsch et al^18^ and Perathoner et al^21^ left the colon stapled-off in situ without reconstruction in 45 of 51 cases. In the remaining, selected cases (6/51), the perforation site was closed with interrupted sutures without colonic resection.^15,18^ Other authors performed colonic resection with colostomy in all cases (HP).^24^ According to Ferrada and Ivatury,^15^ Kwon et al,^16^ and Moore et al,^20^ treatment should be tailored: “a limited colon resection of the inflamed colon is performed using staplers, with no colostomy.” Ferrada and Ivatury,^15^ Kwon et al,^16^ Kafka-Ritsch et al,^18^ and Perathoner et al^21^ preferred temporary abdominal VAC.

Subgroup analysis for recovery in ICU (stage II of DCS) revealed that after surgery, all patients were admitted to ICU for resuscitation. The duration of ICU ranged from 24 to 48 hours in all reports.^15–24^

Subgroup analysis for type of secondary procedures (stage III of DCS) showed that bowel continuity restoration or colostomy was performed by planned relaparotomy^21^ or on-demand relaparotomy^20^ and the choice was based on surgeon's preference.

Moreover, our review failed to determine whether any distinct physiologic parameters (eg, APACHE and Physiological and Operative Severity Score for the Enumeration of Mortality and Morbidity [POSSUM]) were useful to triage patients into receiving DCS versus other treatment.

## DISCUSSION

The goal of DCS is the same as in trauma surgery: the initial emergency operation is to be kept as short as possible and focused on limiting the physiological insult.

The concept of DCS is based on a sequence of key phases^25^: short initial surgery, ICU for resuscitation, and return to the operating room as soon as normal or near-normal physiology is reached for the definitive operation. In trauma patients, this multistage approach is first of all performed to avoid or correct the lethal triad of hypothermia, acidosis, and coagulopathy,^26^ particularly well suited in patients with critical hemodynamic conditions, excessive peritoneal edema, difficulty to obtain a definitive control of the source of sepsis, incomplete debridement of necrotic tissue, uncertainty about bowel viability, uncontrolled bleeding, and massive abdominal wall loss.^27^ The goal of DCS in nontrauma patients is to obtain the same reduction of mortality. Some authors^28^ prefer the terms “advanced open operative treatment of peritonitis” or “aggressive method” terms^29^ instead of DCS. This approach is in accordance with the American Society of Colon and Rectal Surgeons practice parameters for sigmoid diverticulitis: minimum resection and diversion in urgent and emergent cases are generally required.^30^ Perathoner et al^21^ and Kafka-Ritsch et al^18^ underlined the fact that this kind of treatment should be applied only to patients with colic perforation. Others support that laparoscopy can be offered to all patients with complicated diverticulitis ranging from Hinchey stages I–IV.^19^

This review showed that DCS for treatment of peritonitis caused by complicated colon diverticulitis is not always applied according to the same criteria and methods. The results are quite inconsistent, likely because of the innate heterogeneity of patient groups, presentations, and operations. The extreme heterogeneity of indications and procedures make it difficult to analyze this procedure systematically. Nevertheless, it is possible to identify the pros and cons of the different approaches. Although much has been written over the last 50 years, only 2 randomized controlled trial have been performed.^31,32^ In both the studies, one of the arms was a precursor to DCS. Analysis of the published articles demonstrated 2 different surgical approaches, nonetheless not yet compared between themselves. This review showed that DCS is not a new strategy for the management of sepsis caused by diverticulitis, although distinctly different management DCS approaches are presented. Laparoscopy might have a role as DCS only in hemodynamically stable septic peritonitis unresponsive to medical or percutaneous treatment, with lavage/drainage operation but which needs to be validated for both purulent and fecal peritonitis. The question is if inclusion of the concepts, today widely approved, of trauma DCS in the guidelines for diverticulitis, should contribute to standardization of the therapeutic protocols. In the future, there is need to design longitudinal prospective studies apt to prove the results (already obtained for trauma surgery) in the septic complications of AD. The results of 3 ongoing trials are largely awaited (Ladies, Scandiv, and LapLAND trials).^33–35^

## CONCLUSIONS

Our literature review showed that there was an extreme heterogeneity in the performance of DCS for treatment of acute generalized peritonitis caused by sigmoid diverticulitis. Also, the review failed to determine specific physiologic parameters (eg, APACHE, POSSUM, …) that could be used to triage patients to receive DCS versus other treatment. DCS is not a new strategy for the management of sepsis caused by diverticulitis, although distinctly different management DCS approaches are presented. The only new procedure is laparoscopic lavage for perforated diverticulitis (grade III). Clearly, an individual tailored approach to each patient might be appropriate depending on the patient's status.
